# A Case Report on Meningitis Complicated by Acute Ischemic Stroke and Failed Thrombolysis

**DOI:** 10.1002/ccr3.72875

**Published:** 2026-06-05

**Authors:** Sonam Dema, Dechen Choden, Sonam Wangmo

**Affiliations:** ^1^ Department of Internal Medicine Eastern Regional Referral Hospital Mongar Bhutan

**Keywords:** ischemic stroke, meningitis, stroke, thrombolysis

## Abstract

Ischemic stroke is one of the rare but grave complications of meningitis with poor outcomes and long‐term sequelae, and the role of thrombolysis with infection‐related stroke remains uncertain. This case report documents a 52‐year‐old man presented with fever, headache, vomiting, and altered sensorium, and on examination he had neck rigidity. He was treated empirically for bacterial meningitis with antibiotics and dexamethasone, showing initial improvement. But on the 9th day of admission, he developed acute right upper motor neuron hemiparesis, dysarthria, and left‐sided facial palsy. Urgent computed tomography (CT) of the brain showed no hemorrhage, and he was thrombolysed with alteplase within 120 min of symptom onset. Despite timely thrombolysis, there was no neurological recovery. Repeat brain CT confirmed acute ischemic stroke involving the left middle cerebral artery territory. Advanced imaging and endovascular thrombectomy were unavailable. He was managed conservatively with antiplatelets, statins, and physiotherapy, resulting in partial recovery on discharge. Stroke associated with meningitis presents both diagnostic and therapeutic challenges in resource‐limited settings. Thrombolysis in such cases is controversial, and outcomes may be poor due to inflammatory thrombus burden. This case underscores the need for further research and highlights the limitations of stroke management, especially in resource‐limited settings.

**Key Clinical Message**

Meningitis—associated ischemic stroke is a rare but serious complication that poses significant diagnostic and therapeutic challenges, particularly in resource—limited settings where advanced vascular imaging and thrombectomy are unavailable.

## 1 Introduction

The incidence of ischemic stroke in meningitis is variable, ranging from 10%–20% [1], while other studies report 14%–16% [2] in bacterial meningitis cases and a higher incidence around 30% in tuberculous meningitis [3]. In another study, 9%–36% of patients with bacterial meningitis had an ischemic stroke which was associated with a higher in‐hospital mortality, a poor outcome, and long‐term sequelae among survivors compared with patients without stroke [3]. Viruses have also been reported to cause ischemic stroke, mainly varicella zoster virus (VSV), herpes simplex virus (HSV), dengue virus, influenza, and human immunodeficiency virus (HIV), in which about 1%–5% of the patients with HIV had developed a stroke [4, 5].

Meningitis is a serious infection of the meninges, the membranes covering the brain and spinal cord. It can be caused by different pathogens—bacteria, fungi, or viruses. Although viral infections account for most meningitis cases worldwide, the highest global burden in terms of severity, mortality, and long‐term disability is seen with bacterial meningitis [6]. The common cause of community‐acquired bacterial meningitis is *Streptococcus pneumoniae, Neisseria meningitides*, and *Haemophilus influenza type B* [7]. The most frequent presenting symptoms are impaired alertness (78.4%), fever (63.7%), and neck stiffness (51.0%) [3]. Despite the availability of effective antibiotics, bacterial meningitis in adults remains a challenging disease, marked by high mortality and morbidity, particularly in developing countries [7]. According to the World Health Organization (WHO), the South‐East Asia Region (SEAR) accounts for 23% of the deaths due to bacterial meningitis, ranking second highest among the WHO regions [8].

In Bhutan, meningitis shows a significant public health concern, with 217 cases reported annually between 2017 and 2022 [9]. In the year 2022, 32 cases of meningitis were reported among children < 5 years and 9 cases among children between ages of 5–15 years [9]. However, data on adults with meningitis remain scarce [9].

Stroke is a major burden in Bhutan, with Bhutan stroke foundation (BSF) recording 1200 cases annually with 15% mortality in the year 2024 [10]. Since 2023, BSF has been identifying individuals at high risk of stroke, and their findings showed hypertension as the leading cause (73.2%), while other causes of stroke were not included [10].

Eastern Regional Referral Hospital (ERRH) serves as the centre for six districts in the Eastern region of Bhutan. According to ERRH Annual Health report 2024, 57 cases of stroke were managed in the Department of Internal Medicine [11].

Acute ischemic stroke in the setting of meningitis poses diagnostic and therapeutic dilemmas, especially regarding the safety and efficacy of thrombolysis in such patients. We present a case of meningitis complicated by acute ischemic stroke and a failed thrombolysis attempt managed at ERRH.

### 1.1 Case History and Examination

A 52‐year‐old male with no prior comorbidities presented with 5 days history of high‐grade fever associated with headache, nausea, and vomiting with intermittent alteration in mental status characterized by irrelevant verbal responses. Given the patient's fluctuating level of consciousness, the history was obtained from the patient and corroborated by his attendant. He had no similar past episodes and no history of cough, diarrhea, chest pain, weakness of any limbs, or loss of consciousness. He had unremarkable past medical history, past surgical history, and family history. He was not on any regular medications, had no history of allergies, denied history of smoking and illicit drug use (including intravenous drug abuse) but reported a history of drinking alcohol.

On examination, he was febrile with a temperature of 39.1°C, blood pressure (BP) 120/80 mm of Hg, a regular pulse of 82 beats/min, respiratory rate (RR) of 20 breaths/min, and oxygen saturation (SpO2) 94% on room air. He was noted to be altered in consciousness with a Glasgow Coma Scale (GCS) score of 12/15 (E 4 M6 V3) and had neck rigidity suggestive of meningeal irritation, but other signs—Kernig and Brudzinski's were negative. His abdominal, respiratory, and cardiovascular examinations were unremarkable. Neurological examination was difficult to assess due to his altered sensorium; however, cranial nerve examination was normal. His pupils were both equal and reactive, tone in upper and lower limbs was normal, deep tendon reflexes were preserved, and his plantar response was down going.

### 1.2 Differential Diagnosis and Investigation

**FIGURE 1:**
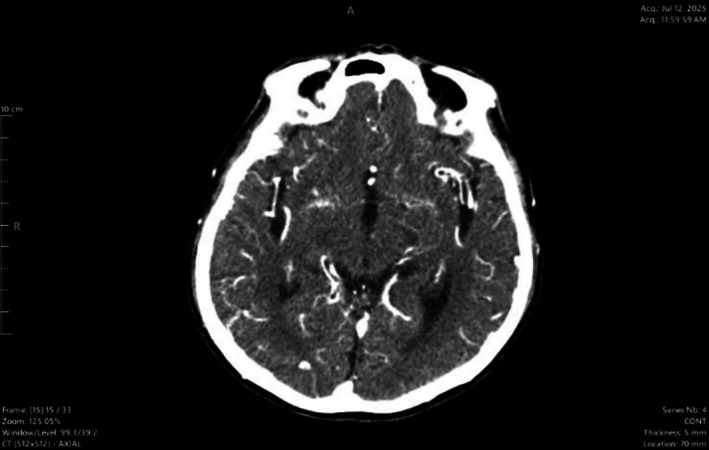
Initial CT Brain done at Emergency—showing Leptomeningeal enhancement.

Based on the history and clinical examination, our initial probable diagnosis was meningitis; he underwent urgent brain CT (Figure 1) which revealed diffuse mild to moderately increased leptomeningeal enhancement, suggestive of—Meningitis. No extra‐axial collection. Electrocardiogram (ECG) showed a normal sinus rhythm, and chest X‐ray was normal.

Laboratory investigation showed leucocytosis (predominantly neutrophilic) with thrombocytopenia of 28 × 10^3/μL (Table 1), along with raised inflammatory markers—Erythrocyte sedimentation rate (ESR) of 80 mm/h, C‐reactive protein (CRP) of 29 mg/L and transaminitis. His renal function test (RFT) and electrolytes were normal. His random blood sugar was 120 mg/dL. His panels for scrub typhus, leptospirosis, and dengue were negative (Table 1) as well as his viral marker. After discussion with the medical specialist, lumbar puncture (LP) was deferred due to thrombocytopenia (28 × 10^3/μL).

**TABLE 1:** Summary of laboratory investigation of 52 years old male with meningitis complicated with ischemic stroke and failed thrombolysis.

Test parameters	At emergency	Day 1	Day 2	Day 4	Day 9 stroke	Day 10	Day 14	Normal value
CBC								
WBC	10.33	7.47	7.47	7.47	8.24	9.20	7.06	4000–10,000cells/cmm
Neutrophils (%)	53.80	68.10	60	63.1	63.50	63	60	
Hemoglobin	10.50	11	11	11	10.60	11	11	13–17 g/dL
Platelets	28	35	55	100	160	172	248	150–450/cmm
LFT								
AST/ALT	102/38	98/29	43/29		26/25	32/17	32/12	5–40 IU/L
Total bilirubin	0.61	0.62	0.62		0.5	0.85	0.59	0–1.20 mg/dL
Indirect bilirubin	0.83	0.37	0.37		0.21	0.57	0.36	0.2–0.8 mg/dL
RFT								
Urea	63	23			40	16	7.24	10–50 mg/dL
Creatinine	1.08	0.74			0.7	0.6	0.73	0.9–1.30 mg/dL
Electrolytes								
Na	139	139			139	138		133–146 mEq/L
K	2.30	2.90			3.40	3.90		3.8–5.40 mEq/L
Ca	8.9							8.80–10.20 mg/dL
PT	15				14.60			13.60–17.5 s
INR	0.9				1.16			0.8–1.20 s
C‐reactive protein	29				12			0–6 mg/L
Scrub Typhus (IgG, IgM)	Negative							
Dengue NS1, IgG, IgM	Negative							
Blood culture	No growth							
Viral marker	Non‐reactive							
Cerebrospinal fluid analysis	Mildly Turbid							
Appearance	Increased							
Pressure	No cells							
WBC	NIL							
Neutrophils	NIL							
Lymphocytes	15 mg/dL							
Protein	52 mg/dL							
Glucose	Negative							
Gram stain	No growth							
Culture	MTB not							
GeneXpert	Detected							

Abbreviations: AST/ALT, Aspartate Aminotransferase/Alanine Aminotransferase; Ca, Calcium; CBC, Complete Blood count; IgM, Immunoglobulin M; IgG, immunoglobulin G; LFT, Liver function Test; MTB, *Mycobacterium tuberculosis* ; Na/K, Sodium/Potassium; NS1, Non‐Structural Protein 1; PT/INR, Prothrombin Time/International Normalized Ratio; RFT, Renal function test; WBC, White Blood Cell.

### 1.3 Management (Treatment)

Based on the 5‐day history of headache and high‐grade fever along with history of alcohol use, with clinical finding of altered sensorium and neck stiffness, and blood investigation revealing leucocytosis with neutrophilic predominance and raised inflammatory markers, the probable diagnosis was meningitis—likely bacterial. Since LP could not be done initially, he was started empirically on IV (Intravenous) antibiotics: Ceftriaxone 2 g 12 hourly, Acyclovir 500 mg every 8 hourly, and Doxycycline 100 mg every 12 hourly along with Dexamethasone 10 mg 6 hourly. He was admitted to the medical ward for continued management and further evaluation.

In medical ward, after Day 2 of antibiotics, he started showing clinical improvement, he was afebrile, his mental status improved (GCS of 15/15) and his headache had resolved. LP was performed on Day 4 of antibiotics, once his thrombocytopenia had improved to platelet count of 100 × 10^3/μL. The cerebrospinal fluid (CSF) report (Table 1) showed mildly turbid in appearance and increase pressure, but showed no cells, no raised protein, and all cultures including CSF GeneXpert were negative which was likely due to the prior antibiotic use.

Based on the patient's clinical presentation of 5 days history of headache, high grade fever, altered sensorium and neck stiffness, along with CT findings showing diffuse leptomeningeal enhancement, elevated inflammatory markers and CSF demonstrating mildly turbid appearance with raised opening pressure, the diagnosis was bacterial meningitis. Although CSF was acellular and culture negative, likely influenced by prior antibiotic use. The patient also showed significant clinical improvement following empirical antibiotic therapy, including resolution of headache and improvement in consciousness and normalization of inflammatory markers, further supporting our diagnosis of bacterial meningitis. Polymerase chain reaction (PCR) and viral panel testing (for *Herpes Simplex Virus Varicella Zoster Virus*) were not available. Fungal and tuberculous meningitis were considered less likely given the short period of history, clinical response and negative tuberculosis workup including his sputum GeneXpert. His fasting, post prandial blood sugars, as well as lipid profile, were normal and his transaminitis had resolved during hospitalization.

On Day 9 of admission at 5:54 pm, he suddenly developed right sided hemiparesis with dysarthria and had left sided upper motor neuron (UMN) facial palsy. According to his attendant, there was no associated chest pain, leg pain nor swelling of legs or history of dyspnoea. At the time of symptom onset, his GCS had dropped to 12/15 (E 4 M6 V3) with vital signs BP of 130/90 mm of Hg, pulse100/min, RR of 20 breaths/min, SPO2 of 95% on room air, temperature 37°C and random blood sugar (RBS) of 100 mg/dL.

**FIGURE 2:**
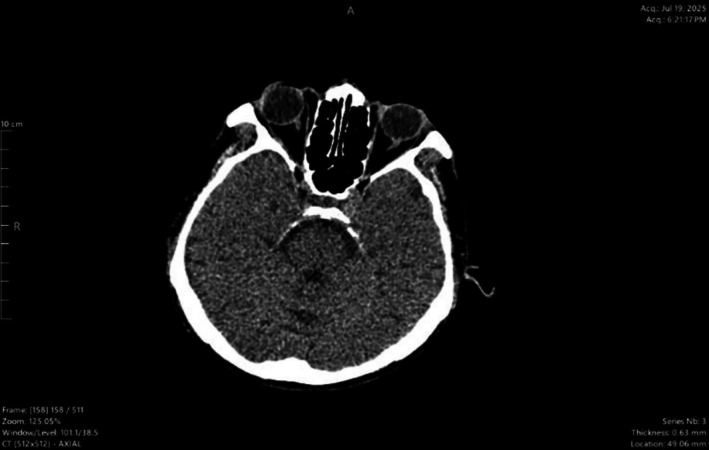
Urgent CT brain done at Day 9 when suspecting in—hospital stroke—showing no infraction or ICH (intracranial hemorrhage).

Suspecting in‐hospital stroke, brain CT (Figure 2) was urgently performed—study reveals no obvious large territorial infarction or acute hemorrhage, generalized cortical atrophy and cerebellum and brainstem are unremarkable. The patient fulfilled criteria for IV thrombolysis after ruling out absolute contraindication, with National Institute of Health stroke scale (NIHSS) of 12/42. His family was updated and after thorough discussion on the pros and cons of thrombolysis, informed consent was obtained. Alteplase was initiated within 120 min of symptom onset. However, despite IV thrombolysis, no neurological improvement was observed after 60 min. Stroke protocol was initiated. He was kept under observation and monitored; his neurological symptoms did not deteriorate further.

**FIGURE 3:**
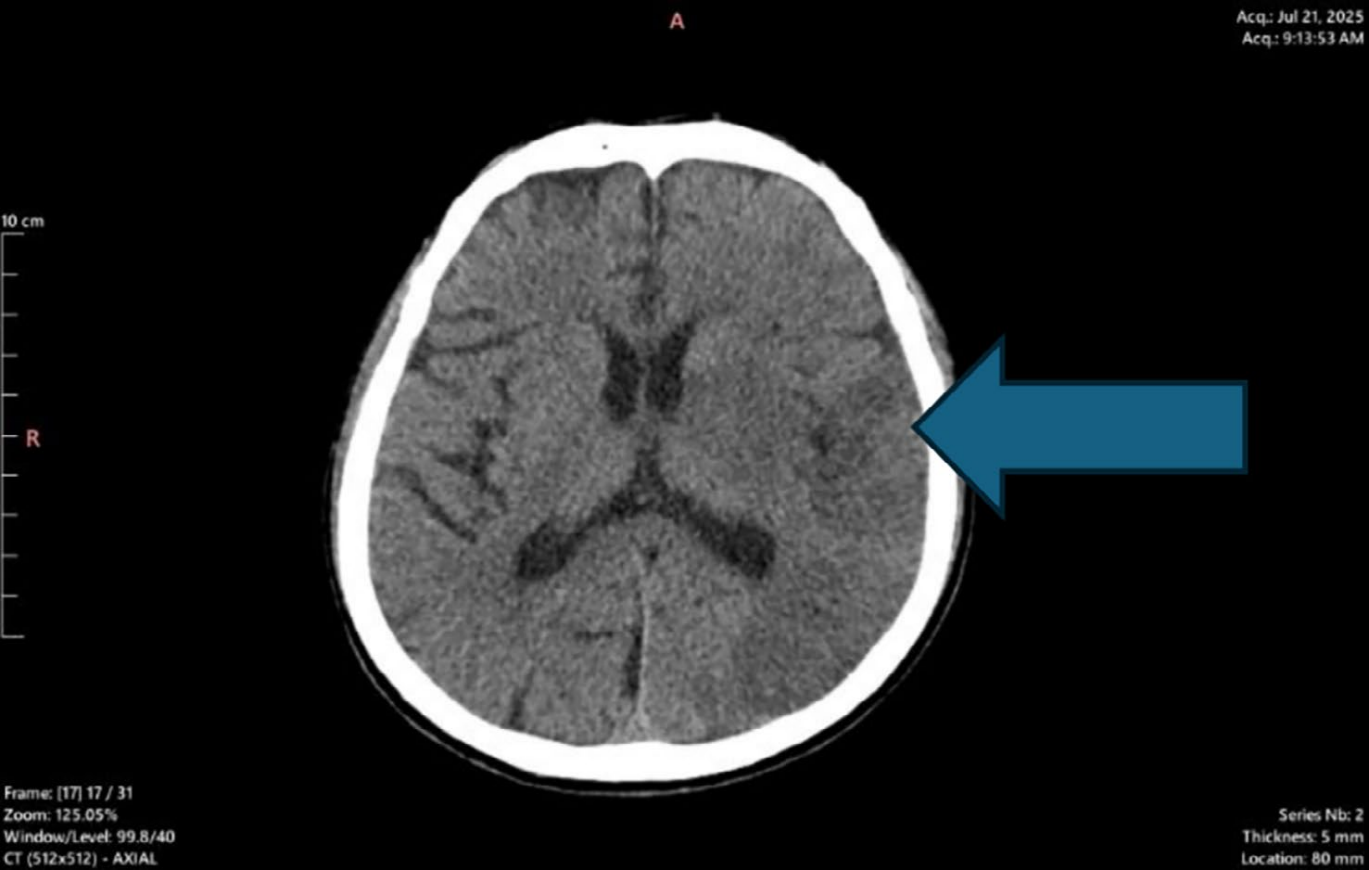
Repeat CT brain done after 15 h of thrombolysis showing left sided ischemic infarct (shown by arrow).

**FIGURE 4:**
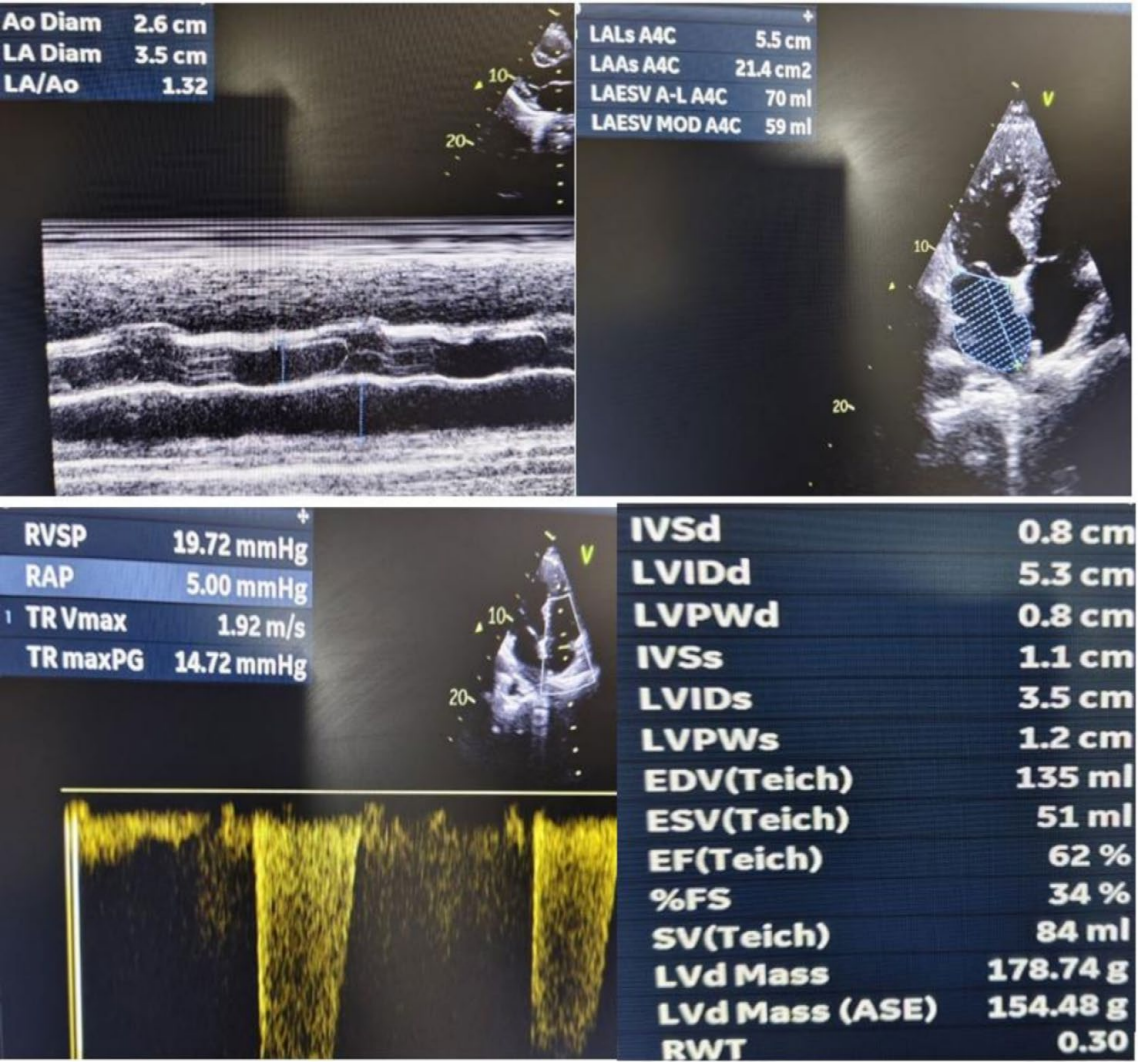
Echocardiogram of 52 years old male with meningitis complicated with ischemic stroke and failed thrombolysis showing—Normal Echocardiogram.

**FIGURE 5:**
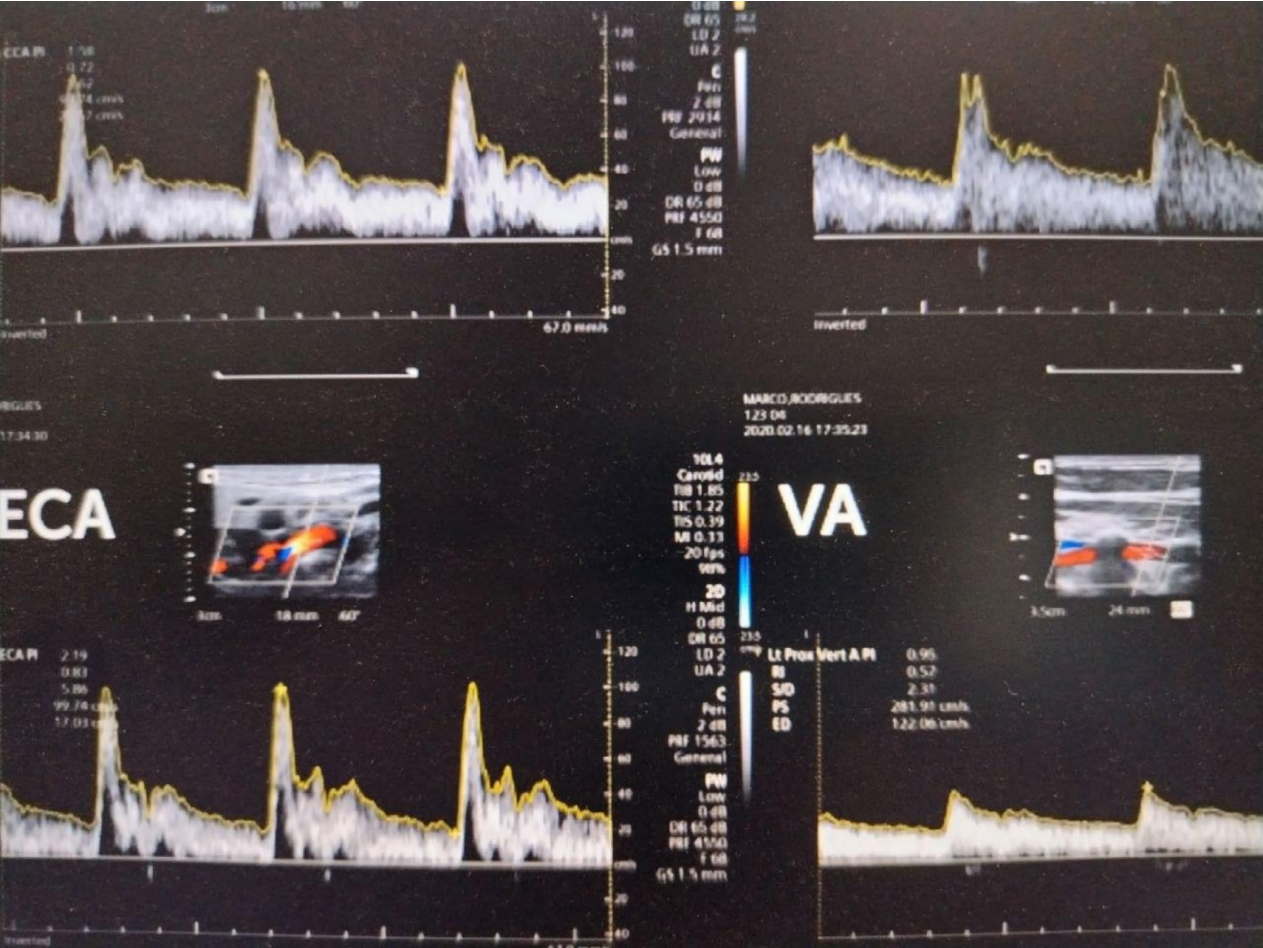
Carotid doppler study of a 52‐year‐old male with meningitis complicated with ischemic stroke and failed thrombolysis showing—Normal carotid artery doppler.

On review the next day, there was no improvement in his neurological deficits. Again, a repeat brain CT was performed which was 15 h after the acute onset of stroke (Figure 3)—which showed interval development of ill‐defined hypodensities at left cerebral hemisphere, left basal ganglia, left internal and external capsules, suggesting acute ischemic stroke and no evidence of acute ICH. Echocardiogram (ECHO) was normal‐ LVEF of 62%, no RWMA, no valvular lesions, no features of patent foramen ovale and no intracardiac shunts/vegetations/thrombus was seen (Figure 4) and carotid doppler (Figure 5) showed bilateral carotid arteries normal in caliber, no intimal thickening or plaques seen, no hemodynamically significant common carotid arteries stenosis. However, Magnetic Resonance Imaging (MRI) with Magnetic Resonance Angiography (MRA) could not be performed due to lack of facilities. His initial work up—fasting blood sugar, post prandial and lipid was normal. Thrombophilia was considered but could not be tested due to lack of facilities. In a young patient without any vascular risk factors, with active central nerve system (CNS) infection and high inflammation suggests that the infection itself may have contributed, mechanism like meningitis‐related vasculopathy/vasculitis or infection‐driven thrombosis.

He was started on single antiplatelet therapy (Aspirin 75 mg) and high intensity statins (Atorvastatin 40 mg), along with stroke care and physiotherapy. Dual antiplatelet therapy was not initiated, given his high NIHSS score of 12/42, and since MRA could not be done to prove large‐artery atherosclerotic disease.

### 1.4 Outcome and Follow‐Up

Antibiotics were administered for 14 days, along with continued stroke care and physiotherapy. After 22 days of hospitalization, he was discharged with residual right‐sided weakness of NIHSS of 4/42, which had improved from 12/42. He was advised to follow up regularly at the local hospital and to continue physiotherapy at home.

## 2 Discussion

This case highlights an uncommon yet important complication of meningitis, an acute ischemic stroke. Our patient had developed an in‐hospital stroke, which is defined as acute infarction of central nervous system tissue that occurs during hospitalization in a patient originally admitted for another diagnosis or procedure [12]. Complications such as stroke has been reported in several studies [3, 13]. Outcomes are typically worse in meningitis patients, since they suffer two insults on top of the primary disease they are already admitted for, leaving them with unfavorable outcomes and long‐term sequelae. In fact, 79% of meningitis patients with stroke had poor outcomes with moderate disability (modified Rankin Score, mRS ≥ 3) compared to 39.8% in patients without a stroke, a statistically significant difference (*p* < 0.01) [3].

Stroke in meningitis is thought to be due to inflammatory vasculitis, thrombosis, or hypercoagulability [12, 13]. Our patient had suffered a left middle cerebral artery (MCA) territory stroke. Reports suggest that the MCA territory is frequently affected due to its anatomical location at the base of the brain, where the inflammatory response is most intense [13]. According to the American Stroke Association, timely intravenous alteplase is recommended to all eligible ischemic stroke patients within 4.5 h, along with secondary treatment with Aspirin [14, 15]. However, thrombolysis in settings of infection or infraction secondary to meningitis remains controversial, and no clear clinical guidelines are available for thrombotic treatment [15].

Our patient had no absolute nor relative contraindication and he had undergone timely thrombolysis with IV alteplase. Despite the intervention, no clinical improvement in his neurological deficits was observed. Potential explanations for failure of thrombolysis [16] in infection‐related stroke have been cited due to heavy inflammatory thrombus burden, thrombus resistance, inadequate drug effect or an unfavorable clot composition. Autopsy studies done in bacterial meningitis patients showed to have demonstrated an extensive inflammation in the meninges, vessel thrombosis and immunoglobulin deposition [16] which may have contributed to the ineffectiveness of the fibrinolytic therapy.

However, in our case, the precise mechanism underlying thrombolysis failure remains uncertain. One possible explanation may be due to an extensive inflammatory process or thrombotic burden associated with the underlying infection. In absence of MRI/MRA imaging at our centre to evaluate vessel patency and characterize the vascular lesion, this mechanism could not be confirmed. Alternative mechanisms should also be considered. Mechanism like persistent large vessel occlusion without recanalization, meningitis‐associated vasculitic stenosis, and micro‐thrombotic disease, all of which have been described in infection‐ related stroke. But further studies are needed to better clarify the mechanisms contributing to thrombolysis failure in such cases.

A retrospective review of patients with in‐hospital stroke receiving IV thrombolysis demonstrated equivalent rates of neurologic improvement compared to those treated for community‐onset strokes [12]. Other studies have also reported reperfusion was successful only in less than 50% of patients [13].

Interestingly, ischemic strokes in meningitis have reported to have occurred in elderly within the first 7 days with peaks at Day 3 and Day 14 [3]. This pattern suggests the possibility that inflammatory vascular mechanisms may contribute to stroke risk and that early adjunctive dexamethasone may have helped suppress this inflammatory response. However, despite its potential benefit in reducing early inflammation, delayed ischemic events have also been described in patients with meningitis, despite the use of steroids, suggesting that inflammation‐related or thrombotic processes may persist [17]. In our patient, he developed ischemic stroke on Day 9 of treatment and he had received earlier a short course of dexamethasone. This delayed event may reflect persistent inflammatory activity or persistent thrombotic burden, although a causal relationship cannot be established. Further studies are needed to better clarify the impact of steroid timing and duration on the risk of ischemic stroke in bacterial meningitis. Earlier vascular imaging, closer neurological surveillance, and optimization of cerebral perfusion might have reduced meningitis‐related stroke; however, their impact on outcome remains uncertain.

Meningitis can be complicated by acute ischemic stroke, and it poses both diagnostic and therapeutic challenges. This case report highlights the rare but important complication in a patient with meningitis and challenges in management and prognosis, especially thrombolysis effectiveness in infection‐related stroke. Despite the thrombolysis, no neurological recovery occurred in our patient.

In resource‐limited settings like Bhutan, lack of advanced imaging and endovascular thrombectomy limits our management. Nonetheless, this case provides valuable insight for optimizing treatment strategies and highlights the urgent need for further research into thrombolysis outcomes in infection‐related stroke.

## 3 Limitations

Our case had several limitations which likely contributed to the outcome. Due to resource constraints, CSF analysis which was performed after initiation of antibiotics may have shown no cells nor growth, and we could not send CSF for viral panels too. The lack of advanced neuroimaging modalities such as MRI along with MRA including access to thrombectomy services may have influenced our management.

## Author Contributions

**Sonam Dema:** conceptualization, data curation, formal analysis, investigation, resources, supervision, visualization, writing – original draft, writing – review and editing. **Dechen Choden:** writing – review and editing. **Sonam Wangmo:** writing – review and editing.

## Funding

The authors have nothing to report.

## Consent

Written informed consent was obtained from the patient for publication of this case report and accompanying images of investigations.

## Conflicts of Interest

The authors declare no conflicts of interest.

## Data Availability

The data is available on request due to privacy/ethical restrictions.
